# NT-020 treatment reduces inflammation and augments Nrf-2 and Wnt signaling in aged rats

**DOI:** 10.1186/s12974-015-0395-4

**Published:** 2015-09-17

**Authors:** Antwoine Flowers, Jea-Young Lee, Sandra Acosta, Charles Hudson, Brent Small, Cyndy D. Sanberg, Paula C. Bickford

**Affiliations:** Department of Neurosurgery Brain Repair, and Center of Excellence for Aging and Brain Repair, University of South Florida Morsani College of Medicine, MDC-78, 12901 Bruce B Downs, Blvd, Tampa, FL 33612 USA; Research Service, James A Haley Veterans Hospital, Tampa, FL USA; School of Aging Studies, University of South Florida, 4202 E. Fowler Ave, Tampa, FL 33620 USA; Natura Therapeutics, Inc., Tampa, FL USA

**Keywords:** NT-020, β-catenin, Wnt pathway, Dentate gyrus subgranular zone, Subventricular zone, Polyphenols, Neuroinflammation

## Abstract

**Background:**

Aging is associated with a decline in stem cell proliferation that is thought to be a result of dysregulated signaling in the neurogenic niche. This results in a diminished and less efficient pool of progenitors. The Wnt pathway plays a key role in the proliferation and differentiation of progenitor cells. Recent publications suggest that the age-related decline in the function of Wnt is a contributor to age-dependent decline in neural progenitors. Similarly, the aged neurogenic niche is characterized by higher levels of inflammatory cytokines. This increased inflammation contributes to the declining function of neural progenitor cells. NT-020, a proprietary blend of polyphenols, has been shown to increase proliferation of neural progenitors and improve cognitive function in aged rats.

**Purpose and methods:**

In this study, we examined the neurogenic niche in the subgranular zone of the dentate gyrus (SGZ) and the subventricular zone (SVZ) of young and aged rats to determine if dietary supplementation with NT-020 could regulate inflammation and oxidative stress response pathways in neurons, astrocytes, and microglia. Further, we examined NT-020’s ability to modulate Wnt signaling in the aged neurogenic niche. To accomplish this, we utilized gene PCR arrays and immunohistochemistry.

**Results:**

We observed an increase in nuclear localization of immunopositive labeling of β-catenin, HO-1, and Nrf2 in all subsets of cell types in both young and aged rats in the SGZ and SVZ following NT-020 treatment. NeuN-positive cells showed a basal increase in nuclear β-catenin in the aged rats, which was not observed in doublecortin (DCX)-labeled cells, microglia, or astrocytes. Reverse transcription polymerase chain reaction (RT-PCR) analysis of isolated hippocampal tissue revealed that a significant percent of genes involved with inflammation are affected by treatment with NT-020. In addition, several genes that regulate Wnt activity were affected by supplementation.

**Conclusions:**

The results suggest that NT-020 activates oxidative stress response pathways and supports pro-neurogenic gene expression in the hippocampus. This may represent the mechanism by which the NT-020 formula enhances performance in learning and memory tasks in aged mice.

**Electronic supplementary material:**

The online version of this article (doi:10.1186/s12974-015-0395-4) contains supplementary material, which is available to authorized users.

## Introduction

As the world’s aging population increases, diseases that disproportionately affect the elderly will take center stage in biomedical research. In addition to increasing disease risk, aging can have deleterious effects on neural plasticity. Impairments in long-term potentiation (LTP), declines in neurogenesis, and synapse dysfunction are observed in the aged hippocampus [1–4]. Interestingly, most of these age-dependent changes overlap with known effects of pro-inflammatory cytokine expression (see reviews [5, 6]). Neuroinflammation is a central component of the aging milieu and contributes to the progression of a number of degenerative diseases, see review [7]. Polyphenolic compounds have shown promise in addressing several aspects of aging including synaptic dysfunction, decreased neurogenesis, and inflammation [8–10]. Here, we demonstrate that a proprietary blend of polyphenolic compounds attenuates inflammatory cytokine expression and enhances pro-neurogenic signaling in the hippocampus.

The hippocampus is unique in its anatomy as it is one of only two areas in the adult brain with an extensive stem cell niche [11]. The role of adult neurogenesis in the formation of new memories and enhancement of learning has been extensively studied over the last decade [2, 12, 13]. One of the major regulators of adult neurogenesis is the Wnt/β-catenin pathway [14]. Proper Wnt signaling is crucial for progenitor proliferation, differentiation, and integration into the granule layer, see review [15–17]. Pathological alterations in Wnt signaling can result in impairments of neurogenesis and behavioral deficits [17, 18]. In addition, Wnt ligands are known to decrease in the aged hippocampus, replacement of which is sufficient to rescue proliferation of neural progenitors in mouse models [17, 19]. While it has been demonstrated that polyphenols like epigallocatechin gallate (EGCG) are capable of enhancing neurogenesis in vivo, no mechanism for this action has yet been proposed. Here, we investigate the interaction between polyphenolic compounds and the Wnt/β-catenin pathway as a possible mechanism by which neurogenesis is enhanced in these models.

The aged hippocampus contains an increased number of activated astrocytes and microglia, as well as increased levels of pro-inflammatory cytokines such as TNF-α and IL-1β [7]. High levels of TNF-α and IL-1β have deleterious effects on neurogenesis, LTP, and synaptic formation [7]. Genomic and proteomic profiles of aged immune cells from the brain have yet to reveal a definitive cause for these changes, but several studies suggest that oxidative stress caused by mitochondrial dysfunction may be a primary contributor [20, 21]. Polyphenols have been shown to have a wide range of neuro-protective effects and may exert their action through modulation of immune cells adaptive stress response system [9, 22]. The nuclear factor erythroid 2-related factor 2 antioxidant response element (Nrf2-ARE) pathway is involved in the cellular response to oxidative stress and leads to the transcription of several antioxidant genes [23]. Loss of Nrf2 increases microglial activation and leads to a primed phenotype similar to that observed with aging [24, 25]. In addition, overexpression of Nrf2 is sufficient to attenuate NF-κB signaling and reduce inflammatory responses [26]. Upregulation of Nrf2-ARE may be a central mechanism by which polyphenols attenuate inflammation [9].

In this manuscript, we utilized PCR arrays and the bioinformatics program Ingenuity Pathway Analysis (IPA) to study the age-dependent changes in the Wnt signaling pathway and expression of cytokines in the hippocampus. We show here that treatment of aged mice with the NT-020 formula decreases expression of inflammatory cytokines, increases expression of anti-inflammatory cytokines and trophic factors, and decreases several antagonists of Wnt signaling. We conclude that dietary supplementation with this formula is sufficient to attenuate the neuro-inflammatory component of aging.

## Materials and methods

### Animals and procedures

Male Fischer 344 rats either young 3 months or 20 months of age were randomly segregated into two groups; one was fed an NIH31 control diet and the experimental group was fed a modified diet which included the NT-020 formulation at 135 mg/kg for 30 days. On day 30, rats were anesthetized deeply with isoflurane gas before euthanasia. All rats were divided into two groups. One group was used for tissue, the hippocampus was removed and used for RNA isolation. Another group was perfused intracardially with 0.1M PBS followed by 4 % paraformaldehyde in 0.1 M phosphate buffer saline and sacrificed; the brain was removed and used for immunohistochemistry. All procedures involving animals were approved by the USF/VA IACUC committee; approved protocol # 4389V.

### Real-time reverse transcription polymerase chain reaction

Tissues were removed and microdissected to isolate the hippocampus. Samples were snap frozen in liquid nitrogen and stored at −80 °C until homogenization. Total RNA was isolated from tissue of adult rat hippocampus using RNeasy mini kit (Qiagen, Valencia, CA, USA). Quantitative reverse transcription polymerase chain reaction (RT-PCR) was performed using DNA Engine Opticon 2© (Bio-rad Hercules, CA) with multi-stage program parameters as follows: 10 min at 95 °C, 40 cycles of 15 s at 95 °C, and 1 min at 60 °C. Samples were tested in triplicate, and the samples obtained from three to five independent tissues were used for the analysis of relative gene expression using the 2 − ΔΔCT method. The following PCR arrays were utilized for this study: WNT signaling targets array PARN-243Z, and cytokine and chemokine array PARN-150Z (Qiagen, Valencia, CA). These arrays contain primers corresponding to 84 genes related to the individual pathways, five housekeeping genes for normalization, and positive and negative controls for the PCR reaction. Genes were considered to be differentially expressed if their expression differed by at least twofold between the young and old or old and old supplemented groups, and that difference was statistically significant across replicates, *p* < 0.05.

### Immunohistochemistry and analysis

Immunohistochemistry was used to identify nuclear labeling of Nrf2 or β-catenin in either neurons (NeuN), microglia (IBA-1), astrocytes (GFAP), or neuronal progenitors (doublecortin (DCX)) observed in young and aged F344 rat brain. The primary antibodies (1:500) were polyclonal antibodies raised in rabbit against NeuN, GFAP, DCX (Abcam), and IBA-1 (Waco) and monoclonal antibodies raised in mouse against β-catenin, Nrf2, or HO-1 (Abcam) with blocking buffer (TritonX-100 (0.02 %), normal goat serum (4 %)). The secondary antiserum (1:500) was Alexa488 anti-mouse and Alexa594 anti-rabbit IgG with blocking buffer. To determine the nuclear co-labeled cells, each field is 550 × 550 μm in area. Total numbers of NeuN, DCX, GFAP, or IBA-1 cells were counted per section, and co-labeling of Nrf2 or β-catenin with DAPI to indicate nuclear co-labeling cells were counted from five different sections from each of six animals. Regions analyzed were the subgranular zone of the dentate gyrus of the hippocampus and the subventricular zone. The nuclear co-labeling with DAPI was confirmed using confocal imaging with 2-μm Z-steps using an FV1000 MPE confocal microscope.

### ELISA

Protein concentration of TNF-α and IL-1β was measured in tissue lysates from hippocampus and cortex using enzyme-linked immunosorbent assay (ELISA) from Raybiotech protein assay. About 100 μl of 100 μg sample concentration was added into appropriate wells and incubated overnight at 4 °C with gentle shaking. The following day, the solution was discarded and the plates were washed four times with 1× wash solution (Raybiotech). Plates were incubated for 1 h at room temperature with detection antibody and washed four times. Of HRP streptavidin solution, 100 μl was added to each well and then incubated for 45 min at room temperature and washed four times. Of TMB (Raybiotech), 100 ul was added to each well and incubated for 30 min at room temperature in the dark. About 50 μl of stop solution (Raybiotech) was added. Absorbance was measured at 450 nm immediately.

## Results and discussion

### NT-020 attenuates inflammation in the aged hippocampus

#### The aged hippocampus is a pro-inflammatory environment

We measured the expression of 84 cytokine and chemokine genes in the hippocampus of young and old (3–5 and 22–25, respectively) rats using the RT-PCR cytokine array from Qiagen PARN-150Z. The aged samples exhibited increased expression of inflammatory genes TNF and IL-1β (Table 1). IL24 is a modulator of immune response associated with the anti-inflammatory alternative activations state of microglia. It was decreased threefold in our data set (Table 1) [27]. These results support previous studies that reported high levels of cytokine and chemokine expression in the serum of aged animals. These results provide a picture of the differential expression of cytokines and chemokines in the aged hippocampus.

**Table 1:** Expression of cytokines and chemokines in aged brain

Gene symbol	Gene name	*p* value	Fold change	Function
Ccl19	Chemokine ligand 19	0.03	1.868	Basal leukocyte migration
Ccl2	Chemokine ligand 2	0.042	1.829	T lymphocytes/monocytes
Ccl3	Chemokine ligand 3	0.003	3.304	Monocytes/macrophages
Ccl4	Chemokine ligand 4	0.03	5.936	Natural killer cells/monocytes
Ccl5	Chemokine ligand 5	0.001	16.009	T cells/eosinophils
Cxcl10	Chemokine ligand 10	0.011	4.271	Multiple
Cxcl11	Chemokine ligand 11	0.001	3.302	T cells
Xcl1	Chemokine ligand 1	0.026	2.331	T cells
Il16	Interleukin 16	0.012	−1.951	CD4+ T cells
Tnf	Tumor necrosis factor	0.029	1.494	Pro-inflammatory
Il1a	Interleukin 1, alpha	0.009	2.458	Pro-inflammatory
Il1b	Interleukin 1, beta	0.006	4.074	Pro-inflammatory
Il15	Interleukin 15	0.009	1.669	Pro-inflammatory
Ltb	Lymphotoxin beta	0.043	1.583	Pro-inflammatory
Il24	Interleukin 24	0.001	−2.925	Anti-inflammatory
Il1rn	Interleukin 1, receptor antagonist	0.001	5.644	Anti-inflammatory

#### NT-020 increases expression of anti-inflammatory cytokines

**Fig. 1:**
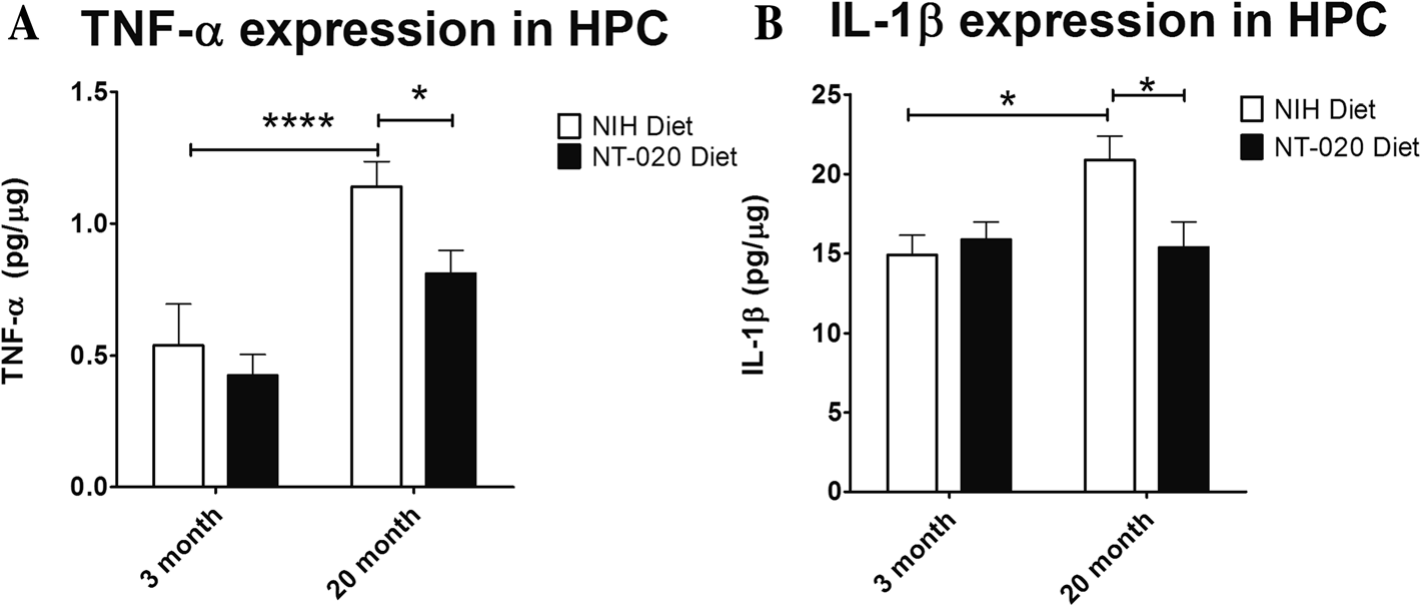
TNF-α and IL-1β protein expression in the CNS as measure by ELISA assay. *Asterisks* indicate significant differences between the groups. **a** Aged rats fed with NT-020-supplemented diet show a 20 % reduction of TNF-α protein expression in the hippocampus in comparison with aged rat fed with control diet. **b** In aged rat fed with NT-020 diet, the IL1-β protein expression in the hippocampus decreased approximately 25 % in comparison with aged rat fed with control diet. There is an overall age-related increase of TNF-α and IL-1β expression in the hippocampus with age. (Two-way analysis of variance (*ANOVA*) in Fig. 2a, *F* = 19.91, degrees of freedom (*df*) = 1, followed by Bonferonni post hoc values (***) *p* < 0.001. In Fig. 2b, *F* = 24.09, *df* = 1, followed by Bonferonni post hoc values (*) *p* < 0.05. In Fig. 2c, *F* = 5.045, *df* = 1, followed by Bonferonni post hoc values (*) *p* < 0.05)

In a previous study of NT-020, aged rats fed an NT-020 diet for 3 weeks had significantly lower numbers of OX-6 (MHC II) positive microglia [28]. Present results confirm that this decreased number of activated microglia is paralleled by a decrease in cytokine and chemokine expression (Table 2).We again analyzed the expression of 84 cytokines and chemokines in the hippocampus of aged rats fed either a control NIH diet or an NT-020 diet. IL24, IL4, and IL10 are cytokines with anti-inflammatory or immune-modulating effects whose expression was increased in the NT-020 diet group (Table 2). Further, expression of CX3CL1 (fractalkine), LIF, and GDNF was also observed (Tables 2 and 4). Expression of these markers is indicative of microglia in an M2 or alternative activation state which supports tissue repair and stem cell proliferation [27, 29]. Expression of the pro-inflammatory cytokine genes IL-1α and IL18 (Table 2) were decreased as was expression of chemokines CCL19 and CCL2 known to be involved in the trafficking of T cells to the CNS [30]. We also performed an ELISA assay to measure levels of both TNF-α and IL-1β. Protein levels were significantly increased in the old hippocampus (Fig. 1). NT-020 treatment decreased protein levels of both TNF-α and IL-1β in 22-month-old rats (Fig. 1). These results suggest that dietary supplementation with NT-020 increases expression of immune-modulatory molecules within the hippocampus. Interestingly as in a previous study [28] with NT-020, this was also accompanied by an improvement in performance on a spatial learning task, in this case, the radial arm water maze in the aged rats (Additional file 1: Figure S1), but not in young rats (Additional file 2: Figure S2).

**Table 2:** Expression of pro-inflammatory cytokines is attenuated by the NT-020 diet

Gene symbol	Gene name	*p* value	Fold change	Process
Lif	Leukemia inhibitory factor	0.018	2.881	Neurotrophin
Gpi	Glucose phosphate isomerase	0.005	1.569	Neurotrophin
Cx3cl1	Chemokine (C-X3-C motif) ligand 1	0.011	1.602	Anti-inflammatory
Cxcl12	Chemokine (C-X-C motif) ligand 12	0.034	1.436	Anti-inflammatory
Il10	Interleukin 10	0.049	1.695	Anti-inflammatory
Il1rn	Interleukin 1 receptor antagonist	0.009	−1.682	Anti-inflammatory
Il18	Interleukin 18	0.033	−1.607	Pro-inflammatory
Il1a	Interleukin 1 alpha	0.011	−2.034	Pro-inflammatory
Il24	Interleukin 24	0.025	1.699	Anti-inflammatory
Ccl19	Chemokine (C-C motif) ligand 19	0.047	−1.506	Chemotaxis
Ccl2	Chemokine (C-C motif) ligand 2	0.047	−1.988	Chemotaxis
Il16	Interleukin 16	0.037	1.798	Chemotaxis

**Fig. 2:**
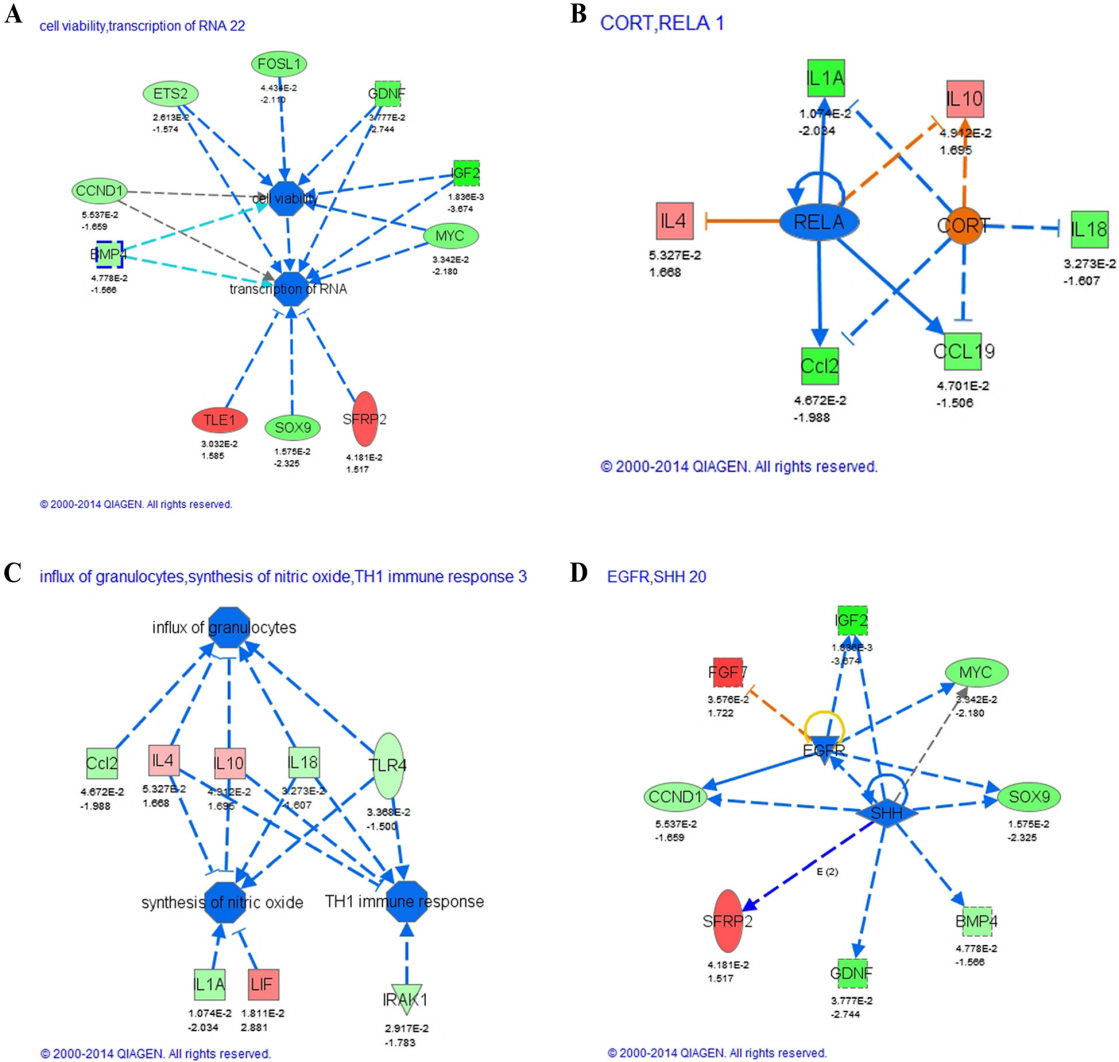
**a** Downstream analysis of Wnt-related gene expression from aged animals predicts a decrease in cell viability and transcription of RNA. **b** Analysis of upstream regulators of cytokine expression in animals supplemented with NT-020 predicts a decrease in the activity of the p65 subunit of NF-κB (RELA) and an increase in corticosterone activity as determined by the concordant activity of their identified downstream targets. **c** Downstream analysis of gene expression predicts a decrease in NO synthesis, TH1 immune response, and influx of granulocytes as a result of the modulation in indicated gene activity. **d** Upstream analysis of the Wnt pathway predicts inhibited activity of Sonic Hedgehog (SHH) and EGF receptor

In an attempt to identify possible regulators upstream of our target molecules, we performed upstream and downstream analysis in the bioinformatics program Ingenuity Pathway Analysis (Fig. 2). The upstream analysis predicts that inhibition of RELA, the p65 subunit of NF-κB, could explain the treatment effect which is consistent with previous reports that EGCG was capable of inhibiting NF-κB in T cells in a model of MS [31]. In addition, there is a predicted increase in the activity of corticosterone (CORT) (Fig. 2b). Low levels and short durations of corticosteroids are known to have anti-inflammatory effects; however, here, it is unlikely that supplementation triggered release of these compounds. Instead, we propose that in the primed state of the aged hippocampus, there may be a blunted response to corticosterone signaling which is restored after supplementation. A recent study examined the potential role of excess glucocorticoids leading to brain aging and AD; a novel finding was observed when comparing the glucocorticoid transcriptome with the aging transcriptome. Although the prevailing hypothesis relates to overactivation of corticosteroids with age, these authors observed that many genes including the inflammatory genes that are upregulated with age are downregulated by cortisol treatments suggesting a downregulation of glucocorticoid responses with age in some cells that could be responsible for the increased inflammatory response with age [32]. Further studies are needed to determine if these upstream regulators are altered in the aged rats with NT-020 treatment. Downstream analysis of bio-functions gives an interpretation of the gene expression data. This analysis is consistent with a decrease in the synthesis of nitric oxide, a major source of oxidative stress in the CNS, the influx of granulocytes, and the TH1 immune response (Fig. 2c). Notably, the genes at the center of these predictions include the anti-inflammatory genes IL4 and IL10. Taking a systematic view of the data has demonstrated that the anti-inflammatory effects of dietary supplementation are broad and involve modulating the activity of multiple cell types in the niche.

#### NT-020 increases nuclear expression of Nrf2 in the dentate gyrus of the hippocampus

**Fig. 3:**
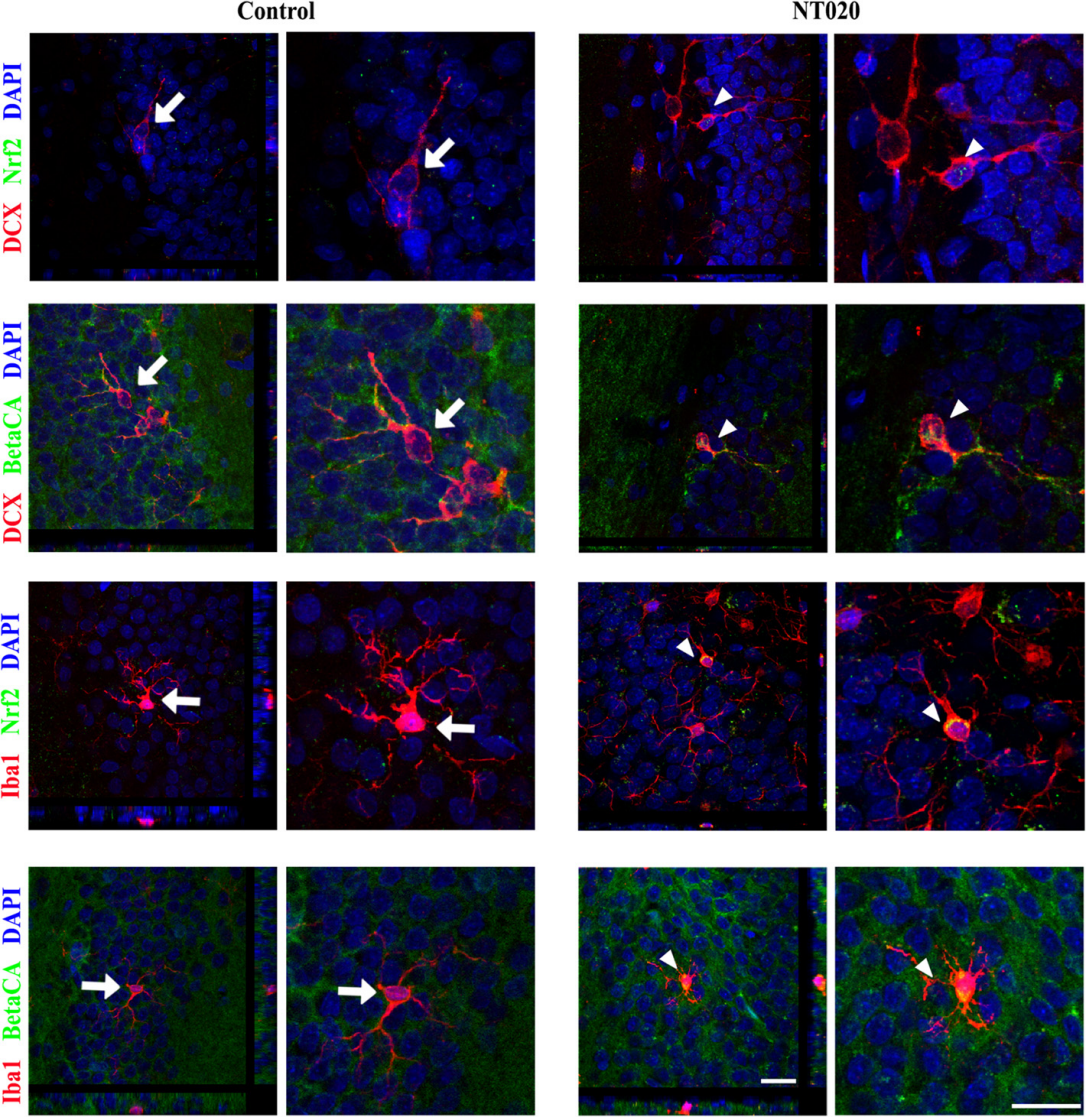
Confocal microscopy was used to examine the nuclear localization of Nrf2, HO-1, and β-catenin. Cellular markers were used to determine specific localization in newborn neurons with doublecortin (*DCX*), astrocytes with GFAP, mature neurons with NeuN, and microglia with IBA-1. Here are shown a few examples of the confocal images demonstrating nuclear (DAPI, *blue*) co-localization of β-catenin or Nrf-2 (*green*) in DCX or IBA-1 (*red*) positive cells in the NT-020-treated aged rats. Z-stacks (1 micron) were taken and rotated in two dimensions as shown in the *side panels* of the figures on the *left* of each subpanel for control and NT-020-treated rats. Higher power images are inserted to the *right* of each image focusing on the cell at the center of the Z-stack rotations. Only cells with clear co-labeling from all three views were counted. Cells shown for control (*large arrows*) clearly show no co-localization. Cells shown in NT-020 panel (*arrowheads*) demonstrate nuclear co-localization

**Fig. 4:**
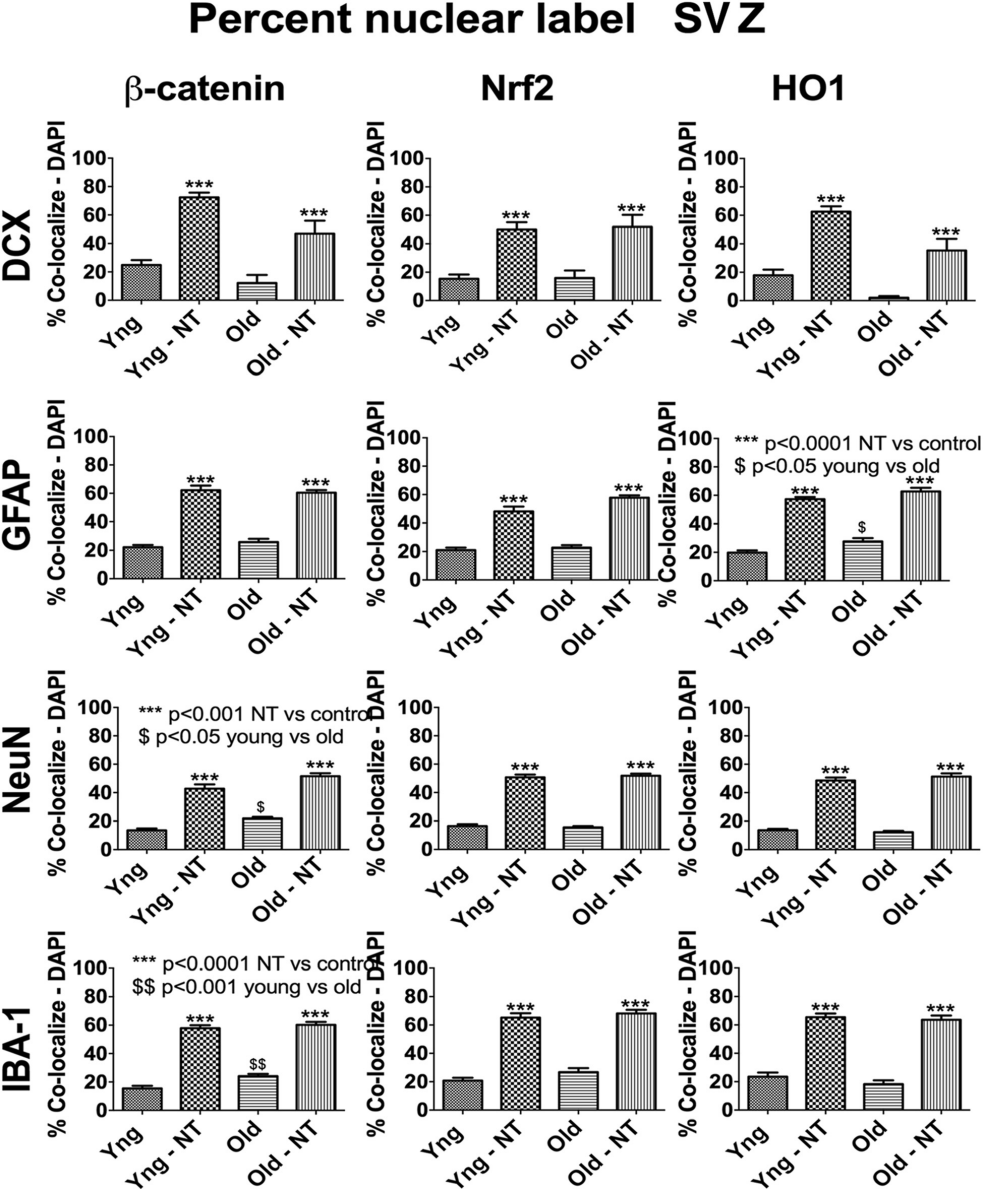
Bar graph summarizing the percent nuclear localization of Nrf2, HO-1, and β-catenin. For each condition, at least 100 cells were counted except for the aged DCX condition where this was not possible due to low cell counts. As can be seen, NT-020 treatment increases the percent of cells with nuclear expression of all three proteins in all four cell types independent of the age of the rats. (One-way ANOVA followed by Tukey’s post hoc analysis ***p < 0.001 for treatment versus age-matched control condition. DCX/β-catenin *F* = 20.63, *df* = 3; DCX/Nrf2 *F* = 12.69, *df* = 3; DCX/HO-1 *F* = 26.87, *df* = 3; GFAP/β-catenin *F* = 86.53, *df* = 3; GFAP/Nrf2 *F* = 65.37, *df* = 3; GFAP/HO-1 *F* = 107.9, *df* = 3; NeuN/β-catenin *F* = 77.1, *df* = 3; NeuN/Nrf2 *F* = 190.7, *df* = 3; NeuN/HO-1 *F* = 165.3, *df* = 3; IBA-1/β-catenin *F* = 144.1, *df* = 3; IBA-1/Nrf2 *F* = 86.3, *df* = 3; IBA-1/HO-1 *F* = 84.16, *df* = 3). Baseline nuclear localization of β-catenin was observed in mature neurons and microglia in aged rats compared with young rats, *p* < 0.05 (Tukey’s multiple comparison test). Basal nuclear localization of HO-1 was only increased in the astrocytes in the aged rats, *p* < 0.05 (Tukey’s multiple comparison test)

Polyphenolic compounds can activate adaptive stress response pathways in the cell, most notably Nrf2-ARE [9]. Because activation of Nrf2 is capable of attenuating the inflammatory response, we investigated NT-020’s ability to drive Nrf2 activation in vivo [26]. Immunohistochemistry was used to examine cellular localization of Nrf-2 and one of the enzymes regulated by Nrf-2, heme oxygenase 1 in the dentate gyrus of the hippocampus. Doublecortin (DCX), IBA-1, NeuN, and GFAP were used to label immature neurons, microglia, neurons, and astrocytes, respectively. Using confocal microscopy, we randomly counted 100 cells per animal and then determined if expression of Nrf-2 was observed in the nucleus (Fig. 3). The percent of cells with nuclear co-localization of Nrf-2 and HO-1 was significantly increased in all four cell types in the rats that received the NT-020 diet (Fig. 4). This increase occurred with treatment in both young and old rats suggesting that these effects are independent of basal inflammation status. This supports that at least some of the polyphenolic activity in vivo comes from an activation of antioxidant response element pathways across most cell types in the CNS. Interestingly, the role of Nrf-2 in microglial priming has become delineated in many studies including a recent study suggesting that Nrf2 is important for phagocytosis [33]. In addition, Nrf-2 is a regulator of CX3CL1 actions, one of the chemokines upregulated by NT-020 treatment reported in Table 2 [34]. Similar results on Nrf-2 and HO-1 were observed in the subventricular zone (SVZ), the other neurogenic-rich niche in the CNS (Additional file 3: Figure S3).

#### NT-020 increases nuclear localization of β-catenin in the dentate gyrus of aged animals

Wnt ligand binding to its receptor triggers an accumulation of intra-cellular β-catenin that correlates with overall Wnt activity [35]. To gain better insight into the activity level of the Wnt pathway, we stained hippocampus sections as above for Nrf2 for nuclear co-localization with β-catenin. The aged group had reduced percent of DCX positive cells with nuclear co-labeled β-catenin although it only approached significance (Fig. 4). However, compared to control, rats on the NT-020 diet have a higher percent of nuclear co-localization of β-catenin in all cell types. This supports the hypothesis that the NT-020 diet increases overall activity of the Wnt pathway in aged animals. Similar results are observed in the SVZ (Additional file 3: Figure S3). To examine this further, we analyzed gene expression of Wnt target genes.

### NT-020 increases expression of Wnt target genes

#### Wnt target gene expression is altered with age

Analysis of Wnt signaling revealed significant changes in genes responsible for regulating both the cell cycle and cell fate decisions (Table 2). The causes of age-related decline in neurogenesis in the hippocampus are still not fully clear, but several studies examining Wnt ligands suggest that the decreased signaling particularly the decrease in the Wnt3a ligand is at least partly responsible for the decline [17]. Wnt target genes associated with cell cycles Cyclin D1, C-Myc, and AHR were decreased by 1.6-, 2.1-, and 2.12-fold, respectively (Table 3). Growth factors GDNF and IGF2 expression were reduced by 2.7- and 3.6-fold, respectively. Extracellular antagonists block ligand access to the FZD receptors preventing signal transduction. We observed increases in Wnt signaling antagonists SFRP2 and TLE1 which were increased by 1.51- and 1.58-fold, respectively (Table 3). Also, DKK1 was also increased but only approached significance. In addition to extracellular antagonists, several co-repressors exist in the nucleus to antagonize TCF/LEF signaling. TLE1 or Groucho is one such repressor that binds TCF and prevents its transcriptional activity in the absence of β-catenin. These results suggest that antagonism of Wnt signaling is a significant factor affecting Wnt signal transduction in the aged brain. Taken together, these alterations paint the picture of a cell environment with a decrease in extracellular growth and survival signals which correlates with the observed effects of aging on neurogenesis.

**Table 3:** Expression of Wnt targets is decreased in aged hippocampus

Gene symbol	Gene name	*p* value	Fold change	Functional gene group
Myc	Myelocytomatosis oncogene	0.033	−2.180	Cell cycle
Gdnf	Glial cell-derived neurotrophic factor	0.038	−2.744	Neurotrophin
Bmp4	Bone morphogenetic protein 4	0.048	−1.566	TGF-β signaling
Igf2	Igf2	0.002	−3.674	Growth factor
Fosl1	Fos-like antigen 1	0.044	−2.110	Transcription factors
Sox9	SRY-box containing gene 9	0.016	−2.325	Transcription factors
Sfrp2	Secreted frizzled-related protein 2	0.042	1.517	Wnt signaling
Tle1	Transducin-like enhancer of split 1)	0.030	1.585	Wnt signaling

#### NT-020 increases expression of pro-neurogenic genes

Gene expression from NT-020-treated rats revealed a complex pattern of gene regulation that may represent a novel mechanism by which these compounds affect neurogenesis (Table 4). Growth factors GDNF and FGF4 levels were increased, both of which support proliferation and survival of young neural progenitors. An increase in NANOG supports the notion that more pluripotent cells are present in treated animals. Paradoxically, expression of the Wnt3a gene was reduced in the supplemented group while nuclear co-localization of β-catenin was increased. One possible explanation is that restoration of Wnt signal transduction by reduction of antagonists SFRP2, TLE1, and WISP1 triggered a negative feedback mechanism. Expression of transcription factor DLK1, a transmembrane member of the Notch family, was increased after supplementation. Studies on DLK1 indicate that it promotes neurogenesis through its modulation of Notch and BMP pathways [36]. Overall, the gene expression changes are consistent with increased WNT downstream signaling. We postulate that this is a result of reduced expression of negative regulators. These results put forward several possible mechanisms by which NT-020 rescues neurogenesis in the aged niche, and further analysis is necessary to understand what role these changes play in attenuation of the aged phenotype.

**Table 4:** NT-020 diet increases expression of genes involved in regulating self-renewal

Gene symbol	Gene name	*p* value	Fold change	Functional gene group
Gdnf	Glial cell-derived neurotrophic factor	0.020	2.231	Neurotrophin
Dlk1	Delta-like 1 homolog (*Drosophila*)	0.023	4.728	Differentiation and development
Id2	Inhibitor of DNA binding 2	0.002	−1.569	Differentiation and development
Fgf4	Fibroblast growth factor 4	0.015	1.899	Growth factor
Nanog	Nanog homeobox	0.016	1.809	Transcription factors
Sfrp2	Secreted frizzled-related protein 2	0.001	−2.685	Wnt signaling
Tle1	Transducin-like enhancer of split 1 (E(sp1) homolog, *Drosophila*)	0.006	−2.000	Wnt signaling
Wisp1	WNT1-inducible signaling pathway protein 1	0.007	−2.166	Wnt signaling
Wnt3a	Wingless-type MMTV member 3A	0.038	−5.209	Wnt signaling

## Conclusion

The impact of age-dependent inflammation in the CNS is known to drive the progression of neurodegenerative disease and contribute to the decline of tissue homeostasis even in the absence of an underlying disease. The negative impact of inflammatory cytokines on processes vital for cognitive function has been well documented (see review [7]). While it is not fully understood how declining neurogenesis directly impairs learning, it has been shown to be an early event in the pathology of several diseases including Alzheimer’s [2]. This report describes for the first time an increase in Wnt antagonists in the aged hippocampus and their attenuation by supplementation with NT-020. It is not clear whether this occurs as a direct result of the associated attenuation of inflammation and increase in Nrf2-ARE pathways or by an independent mechanism. However, signaling in the niche rarely occurs unilaterally, and it is likely that these compounds act on supporting cells such as astrocytes or microglia in addition to actions directly on the neural progenitor cells themselves. Another finding that may be of particular importance in the NT-020 treatment group is an increase in anti-inflammatory chemokines CX3CL1 and a decrease in chemokines such as CCL19 and CCL2 that are upregulated during periods of inflammation and facilitate the invasion of peripheral leukocytes [30]. While we did not directly measure leukocyte invasion or blood-brain barrier permeability, it is known to be altered with age [37] and it would be interesting to examine the effectiveness of NT-020 supplementation on these parameters in disease models involving chronic inflammation.

It is clear that an age-related increase in microglial priming has direct actions on the neurogenic niche. Aged microglia when co-cultured with neural progenitors reduces stem cell proliferation [38]. It has been suggested that this impairment of neurogenic niche function is driven, at least in part, by Nrf2 [39]. Treatments that increase Nrf2 reduced microglial priming and reduced the effect of aged microglia to inhibit neural progenitor proliferation [39]; interestingly, these authors also suggested that this was associated with increased Wnt signaling [39], all of which were observed in this study. Our findings demonstrate increased Nrf2 translocation to the nucleus after NT-020 treatment and an increase in HO-1, one of the enzymes regulated by this transcription factor. Nrf-2 has been shown to protect neurons from increased oxidative stress during periods of inflammation [26]. Further, Nrf-2 plays a role in microglial priming as the absence of Nrf2 changes microglial phenotype towards that of increased M1 [24], the primed microglial phenotype that is observed with normal aging [25]. Recent evidence linking Nrf2-ARE to regulation of immune cells include that Nrf2-ARE is essential for promoting phagocytosis of phosphorylated tau by microglia/macrophages as shown by a reduction in phagocytosis using a Nrf2 decoy [33]. In addition, increased expression of Nrf-2 has been shown to attenuate inflammation by blocking NF-κβ activity [40].

Taken together, our results demonstrate that NT-020 at concentrations attainable through dietary supplementation effectively target multiple processes that contribute to cognitive decline during aging as well as progression of neurodegenerative disease.

## Additional files

Additional file 1: Figure S1. Performance on the radial arm water maze of aged (21 months old) rats across days. A learning criterion was set to 1 error as shown with the dashed line. As can be observed, aged rats reach criterion by day 4. Performance of old rats on trials 1 and 2 of the second day of training was improved by NT-020 treatment. There was also improved performance during reversal training of radial arm water maze (unpaired *t* test aged rats control vs. aged rats treatment trial 1 of day 2 *p* = 0.0413, trial 2 of day 2 *p* = 0.0399, trial 3 of RT *p* = 0.0317). (TIF 167 kb) Additional file 2: Figure S2. Performance on the radial arm water maze of young (4 months old) rats across days. A learning criterion was set to 1 error as shown with the dashed line. As can be observed, young rats reach criterion by day 3 of training. Treatment of young rats with NT-020 did not alter performance during training, although there was a difference during the probe trial, but not the reversal training. (Two-way ANOVA with repeated measures revealed aging group differences). (TIF 248 kb) Additional file 3: Figure S3. Confocal microscope was used to examine the nuclear localization of Nrf2, HO-1, and β-catenin. Four cellular markers were used to determine specific localization in newborn neurons with doublecortin (DCX), astrocytes with GFAP, mature neurons with NeuN, and microglia with IBA-1. For each condition, at least 100 cells were counted except for the aged DCX condition where this was not possible due to low cell counts. As can be seen, NT-020 treatment increase nuclear expression of all three proteins in all four cell types independent of the age of the rats. One-way ANOVA followed by Tukey’s post hoc analysis ****p* < 0.001 for treatment versus age-matched control condition DCX-Nrf2 *F* = 26.96, *df* = 3 ; DCX-HO1 *F* = 10.70, *df* = 3; DCX-BetaCa *F* = 64.89, *df* = 3; GFAP-Beta-CA *F* = 89.82, *df* = 3; GFAP-Nrf-2 *F* = 125.9, *df* = 3; GFAP-HO1 *F* = 87.81, *df* = 3; NeuN-Nrf2 *F* = 72.16, *df* = 3; NeuN-BetaCa F = 66.13, *df* = 3; NeuN-HO1 *F* = 77.43, *df* = 3; IBA1-BetaCa *F* = 48.96, *df* = 3; IBA-1Nrf2 *F* = 28.64, *df* = 3; IBA1-HO1 *F* = 34.78, *df* = 3). (TIF 375 kb)
