# Erratum to: NT-020 treatment reduces inflammation and augments Nrf-2 and Wnt signaling in aged rats

**DOI:** 10.1186/s12974-016-0669-5

**Published:** 2016-09-05

**Authors:** Antwoine Flowers, Jea-Young Lee, Bethany Grimmig, Sandra Acosta, Charles Hudson, Brent Small, Cyndy D. Sanberg, Paula C. Bickford

**Affiliations:** 1 Department of Neurosurgery Brain Repair, and Center of Excellence for Aging and Brain Repair, University of South Florida Morsani College of Medicine, MDC-78, 12901 Bruce B Downs, Blvd, Tampa, FL 33612 USA; 2 Research Service, James A Haley Veterans Hospital, Tampa, FL USA; 3 School of Aging Studies, University of South Florida, 4202 E. Fowler Ave, Tampa, FL 33620 USA; 4 Natura Therapeutics, Inc., Tampa, FL USA

## Erratum

Upon publication of the original article [1], it was noticed that Bethany Grimmig was missed off the author list. She has now been included and this has been updated in the original article.
