# Supplementary figures and images for: NT-020 treatment reduces inflammation and augments Nrf-2 and Wnt signaling in aged rats
Source: J Neuroinflammation. 2015 Sep 17;12:174. doi: 10.1186/s12974-015-0395-4 (PMC4574066; doi:10.1186/s12974-015-0395-4)

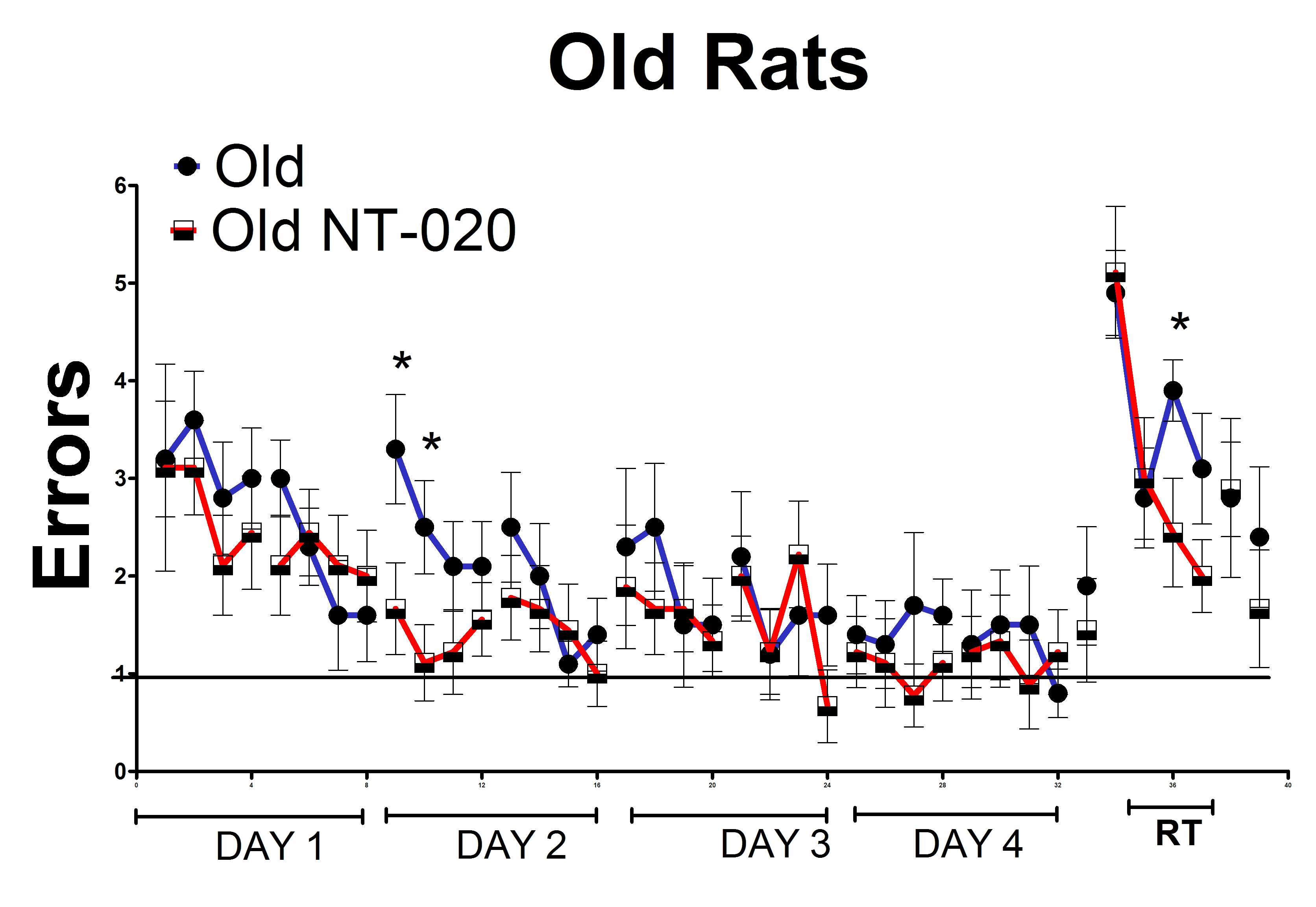

Supplement: Additional file 1: Figure S1. — Performance on the radial arm water maze of aged (21 months old) rats across days. A learning criterion was set to 1 error as shown with the dashed line. As can be observed, aged rats reach criterion by day 4. Performance of old rats on trials 1 and 2 of the second day of training was improved by NT-020 treatment. There was also improved performance during reversal training of radial arm water maze (unpaired t test aged rats control vs. aged rats treatment trial 1 of day 2 p = 0.0413, trial 2 of day 2 p = 0.0399, trial 3 of RT p = 0.0317). (TIF 167 kb) [file 12974_2015_395_MOESM1_ESM.tif]

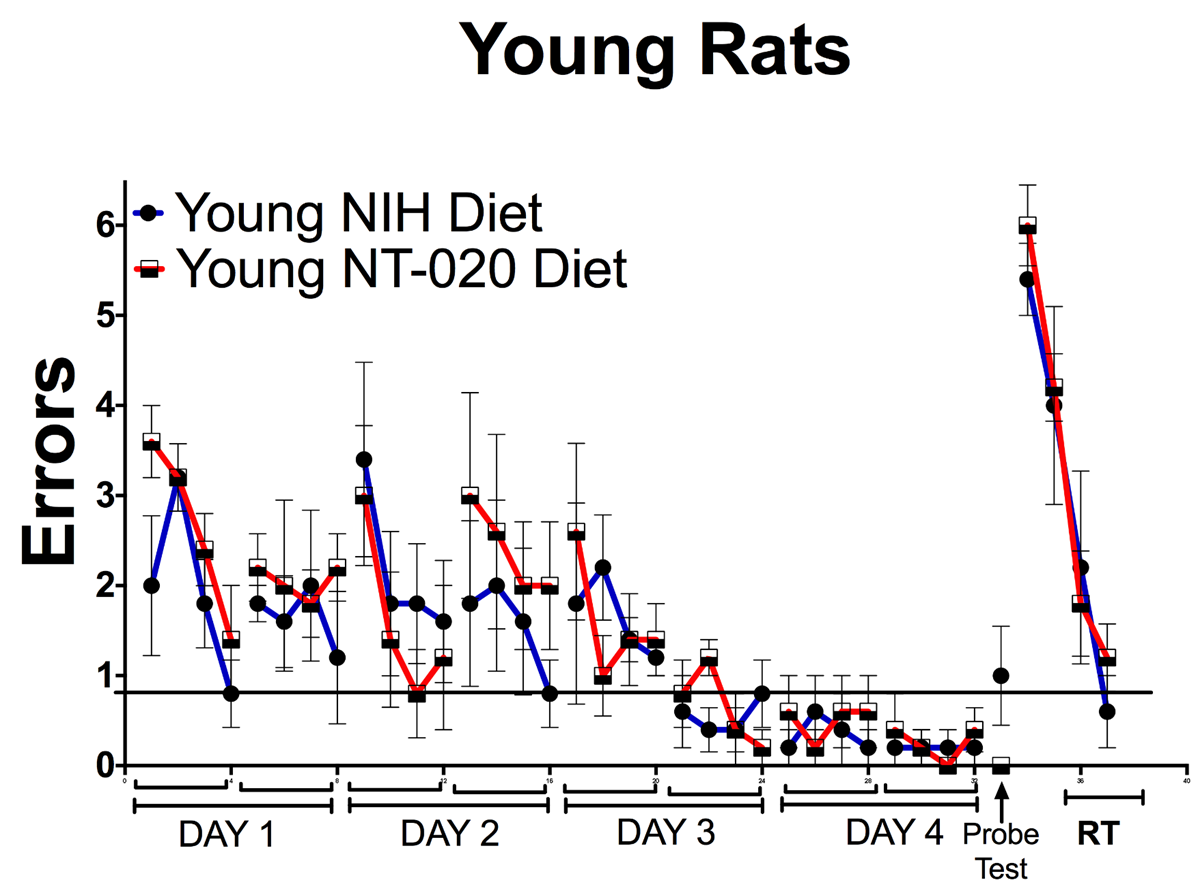

Supplement: Additional file 2: Figure S2. — Performance on the radial arm water maze of young (4 months old) rats across days. A learning criterion was set to 1 error as shown with the dashed line. As can be observed, young rats reach criterion by day 3 of training. Treatment of young rats with NT-020 did not alter performance during training, although there was a difference during the probe trial, but not the reversal training. (Two-way ANOVA with repeated measures revealed aging group differences). (TIF 248 kb) [file 12974_2015_395_MOESM2_ESM.tif]

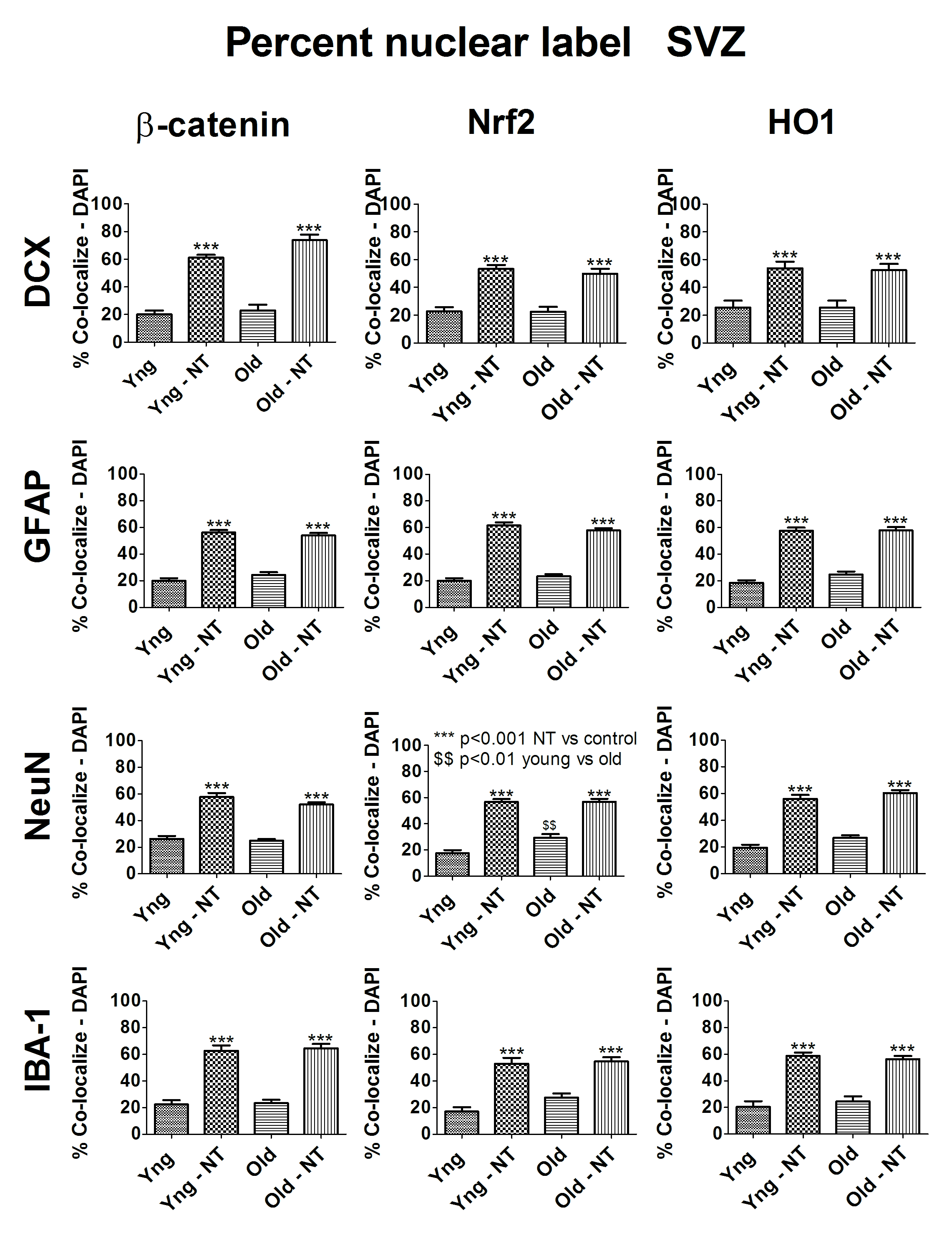

Supplement: Additional file 3: Figure S3. — Confocal microscope was used to examine the nuclear localization of Nrf2, HO-1, and β-catenin. Four cellular markers were used to determine specific localization in newborn neurons with doublecortin (DCX), astrocytes with GFAP, mature neurons with NeuN, and microglia with IBA-1. For each condition, at least 100 cells were counted except for the aged DCX condition where this was not possible due to low cell counts. As can be seen, NT-020 treatment increase nuclear expression of all three proteins in all four cell types independent of the age of the rats. One-way ANOVA followed by Tukey’s post hoc analysis ***p < 0.001 for treatment versus age-matched control condition DCX-Nrf2 F = 26.96, df = 3 ; DCX-HO1 F = 10.70, df = 3; DCX-BetaCa F = 64.89, df = 3; GFAP-Beta-CA F = 89.82, df = 3; GFAP-Nrf-2 F = 125.9, df = 3; GFAP-HO1 F = 87.81, df = 3; NeuN-Nrf2 F = 72.16, df = 3; NeuN-BetaCa F = 66.13, df = 3; NeuN-HO1 F = 77.43, df = 3; IBA1-BetaCa F = 48.96, df = 3; IBA-1Nrf2 F = 28.64, df = 3; IBA1-HO1 F = 34.78, df = 3). (TIF 375 kb) [file 12974_2015_395_MOESM3_ESM.tif]
